# A Leaf-Mimicking Method for Oral Delivery of Bioactive Substances Into Sucking Arthropod Herbivores

**DOI:** 10.3389/fpls.2020.01218

**Published:** 2020-08-11

**Authors:** Noureldin Abuelfadl Ghazy, Mayo Okamura, Kanae Sai, Sota Yamakawa, Faten Abdelsalam Hamdi, Vojislava Grbic, Takeshi Suzuki

**Affiliations:** ^1^ Graduate School of Bio-Applications and Systems Engineering, Tokyo University of Agriculture and Technology, Koganei, Japan; ^2^ Agriculture Zoology Department, Faculty of Agriculture, Mansoura University, El-Mansoura, Egypt; ^3^ Japan Society for the Promotion of Science, Chiyoda, Japan; ^4^ Department of Biology, The University of Western Ontario, London, ON, Canada; ^5^ Instituto de Ciencias de la Vid y el Vino, Logrono, Spain; ^6^ Institute of Global Innovation Research, Tokyo University of Agriculture and Technology, Koganei, Japan

**Keywords:** aphids, artificial diet, membrane feeding assay, spider mites, RNAi

## Abstract

Spider mites (Acari: Tetranychidae) are pests of a wide range of agricultural crops, vegetables, and ornamental plants. Their ability to rapidly develop resistance to synthetic pesticides has prompted the development of new strategies for their control. Evaluation of synthetic pesticides and bio-pesticides—and more recently the identification of RNA interference (RNAi) target genes—requires an ability to deliver test compounds efficiently. Here we describe a novel method that uses a sheet-like structure mimicking plant leaves and allows for oral delivery of liquid test compounds to a large number of individuals in a limited area simultaneously (~100 mites cm^−2^). The main component is a fine nylon mesh sheet that holds the liquid within each pore, much like a plant cell, and consequently allows for greater distribution of specific surface area even in small amounts (10 µl cm^−2^ for 100-µm mesh opening size). The nylon mesh sheet is placed on a solid plane (*e.g.*, the undersurface of a Petri dish), a solution or suspension of test compounds is pipetted into the mesh sheet, and finally a piece of paraffin wax film is gently stretched above the mesh so that the test mites can feed through it. We demonstrate the use of the method for oral delivery of a tracer dye (Brilliant Blue FCF), pesticides (abamectin and bifenazate), dsRNA targeting the *Vacuolar-type H^+^-VATPase* gene, or fluorescent nanoparticles to three species of *Tetranychus* spider mites (Acari: Tetranychidae) and to the cotton aphid, *Aphis gossypii* Glover (Hemiptera: Aphididae). The method is fast, easy, and highly reproducible and can be adapted to facilitate several aspects of bioassays.

## Introduction

Effective delivery of xenobiotics to herbivorous arthropods is a prerequisite for evaluating synthetic pesticides and bio-pesticides. The delivery method should be simple and reproducible and allow for precise estimation of the lethal concentration of a candidate agent (Kabir et al., 1993). Several methodologies are used for evaluating synthetic pesticides against spider mites (Acari: Tetranychidae), including direct spraying, leaf-dip, slide-dip, residual contacted vial, and membrane feeding bioassays (*e.g.*, Potter, 1952; Dittrich, 1962; Hanna and Hibbs, 1970; Kwon et al., 2010).

Pesticide spraying—usually using the Potter spray tower that simulates field application (Potter, 1952)—is probably the most common method of assessing toxicity and resistance in mites. This method, however, requires costly laboratory equipment and a large liquid volume. In the leaf-dip method, an infested or uninfested plant leaf is dipped into a test solution for about 5 s and then air dried (*e.g.*, Dittrich, 1962; Knight et al., 1990). A major source of variability is the uneven distributions of the residues and test mites on the dipped leaf in addition to the high probability of mite escape from the treated leaf. In the slide-dip method, mites are affixed on double-sided adhesive tape attached to a glass slide, and then the whole set is dipped into a test solution for several seconds (Dittrich, 1962). Both the leaf-dip and slide-dip methods share the common drawback of requiring a large volume of test solution. Moreover, according to Dittrich (1962), when using the slide-dip method, about 2 h of preparation is needed to affix *ca.* 60 mites onto 10 slides. The residual contacted vial method is used to assay pesticide resistance in field populations (Kwon et al., 2010); the inner surface of a 5-ml glass vial is coated with 100 µl of an acetone-based test solution and kept for about 1 h under a fume hood until the acetone is completely dried. Although the preparation is simple because plant material is not required, mite handling and mortality scoring after treatment, particularly under non-lethal concentrations, can be problematic. The membrane feeding method was originally developed as a feeding device for the beet leafhopper, *Citculifer tenellus* Baker (Hemiptera: Cicadellidae); artificial diet was placed in a sachet made of fish-skin membrane so that the test insects could feed through it (Carter, 1927). Mittler and Dadd (1962) later developed a membrane feeding device using an extensible and waterproof paraffin wax film (*i.e.*, Parafilm) for the green peach aphid, *Myzus persicae* (Sulzer) (Hemiptera: Aphididae). Using the Parafilm membrane feeding method, many nutritional and pharmacological studies have been carried out on aphids, spider mites, planthoppers, thrips, bedbugs, whiteflies, and mosquitoes (Dadd and Mittler, 1966; Walling et al., 1968; Mitsuhashi and Koyama, 1969; Hanna and Hibbs, 1970; van der Geest et al., 1983; Montes et al., 2002; Gotoh et al., 2008; Upadhyay et al., 2011; Costa-da-Silva et al., 2013; Torres-Quintero et al., 2013; Suzuki et al., 2017a). Although the membrane feeding method was successfully used, the small specific surface area of the feeding arena that coincides with the liquid under the membrane limits the efficiency of the bioassays. Overall, these methods are not suitable for the delivery of extracts/compounds that are available in small amounts and, with the exception of spraying, are less feasible for testing pesticide toxicity on mite immature stages.

In addition to the delivery of chemical compounds, there is also a need for efficient delivery of double-stranded RNA (dsRNA) for RNA interference (RNAi)-based functional genomics and pest management. To date, the dsRNA is delivered into mites using three common methods: microinjection, soaking, or orally *via* plant leaf discs. Microinjection is widely used for delivering dsRNA into nematodes and insects (Fire et al., 1998; Bucher et al., 2002). Although microinjection was used for dsRNA delivery to the two-spotted spider mite, *Tetranychus urticae* (Acari: Tetranychidae) (Khila and Grbić, 2007), this method is not feasible for practical application due to the difficulty of injecting mites that are ~0.5 mm in length and the possibility of causing physical damage (Price and Gatehouse, 2008; Yu et al., 2013; Suzuki et al., 2017a). Delivery of dsRNA *via* soaking mites in dsRNA solution was also demonstrated, but the difficulties of recovering mites after soaking and soaking of immature stages reduce the utility of this method for high-throughput RNAi screens (Suzuki et al., 2017a; Suzuki et al., 2017b). Ultimately, delivering dsRNA orally *via* feeding is the most attractive method because it is the least invasive (*i.e.*, entails no physical damage to test organisms) and is conducive to dsRNA application as a bio-pesticide.

The leaf disc-mediated oral delivery of dsRNA (*i.e.*, foliar application) is widely used for triggering RNAi in spider mite species. Kwon et al. (2013) used leaf discs floating on a dsRNA solution to orally deliver dsRNA to mites. This method requires a large volume of dsRNA solution and consequently a large amount of dsRNA. Leaf disc dehydration is a modified method of foliar application of dsRNA used for the carmine spider mite, *Tetranychus cinnabarinus* (Boisduval), where plant leaf discs are dehydrated at 60°C for 3 min and then soaked in a dsRNA solution for 5 h (Shi et al., 2015). Thereafter, the surface-dried leaf discs are desiccated and used for the bioassay. Broad application of this method is uncertain, however, because the preparation of leaf discs requires a long time, and different plant species may vary in their tolerance to high-temperature dehydration. More recently, Suzuki et al. (2017b) have used the leaf coating approach to deliver dsRNA into *T. urticae*. Although this method uses a small volume (~7.6 μL cm^−2^ leaf disc) of dsRNA solution compared to the floating leaf discs, it requires the manual spreading of dsRNA in order to cover an entire leaf surface. Alternatively, a surfactant such as Silwet L-77 can be used to promote liquid dispersion, but it has been shown to have a negative impact on some test organisms (Cowles et al., 2000; Ray and Hoy, 2014; Abouelmaaty et al., 2019). Hence, identification of a high-throughput dsRNA delivery method to mites remains a great challenge.

We recently developed the mesh method for delivering a test solution or suspension to sucking arthropods. This method allows easy manipulation of test arthropods, uses a small liquid volume, requires no plant material, and the overall preparation requires no specialized skills. Here, we demonstrate the use of the mesh method to deliver tracer dye (Brilliant Blue FCF), pesticides (abamectin and bifenazate), dsRNA targeting the *Vacuolar-type H^+^-VATPase* gene, and fluorescent nanoparticles to TSSM. In addition, we also delivered the tracer dye to tomato red mite, *Tetranychus evansi* Baker and Pritchard, Kanzawa spider mite, *Tetranychus kanzawai* Kishida, and cotton aphid, *Aphis gossypii* Glover (Hemiptera: Aphididae), to test the flexibility of our method. We anticipate that this method will enable large-scale, high-throughput screens of active ingredients of synthetic pesticides and bio-pesticides that include environmental RNAi-based pesticides in spider mites and other sucking herbivores.

## Materials and Methods

### Mite and Aphid Colonies

The reference population of *T. urticae* was established in early 2000 and has previously been used for whole-genome sequencing (Grbić et al., 2011). The *T. urticae* population is maintained in the laboratory on seedlings of kidney beans (*Phaseolus vulgaris* L.) at an air temperature of 25°C, relative humidity of 50%, and light period of 16 h day^−1^. The population of *T. evansi* was collected from black nightshade (*Solanum nigrum* L.) in Tokyo, Japan, in 2006 (Gotoh et al., 2009). The *T. evansi* population is maintained in the laboratory on detached leaves of eggplant (*Solanum melongena* L. cv. Senryo #2). The *T. kanzawai* population was collected from red clover (*Trifolium pratense* L.) in Sobetsu, Hokkaido, Japan in 2008 and is routinely reared on detached leaves of *P. vulgaris*. The population of *A. gossypii* is maintained in the laboratory on detached leaves of *P. vulgaris*. An air pump-based system (Cazaux et al., 2014; Suzuki et al., 2017a) was used to collect mites or aphids used in the following experiments.

### Preparation of the Feeding Device in the Mesh Method

**Figure 1:**
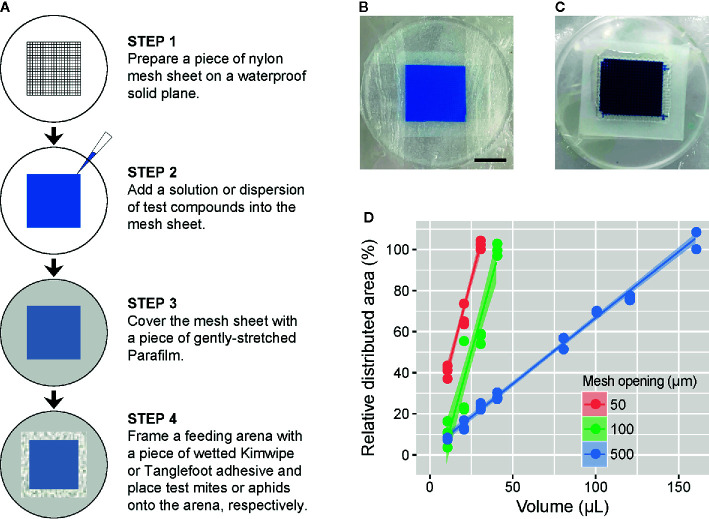
Description of the feeding device in the mesh method. **(A)** Schematic of the procedure for preparation of the feeding device in the mesh method. Prepared feeding device for **(B)** mites and **(C)** aphids, in which a 1 or 2.5% (w/v) blue tracer dye (Brilliant Blue FCF) was added into the nylon mesh sheet (2 × 2 cm) with a 100- or 500-µm opening size, respectively. Scale bar: 1 cm. **(D)** The relationship between the volume of tracer dye solution and the solution-filled area of the nylon mesh sheet (2 × 2 cm) with a 50-, 100-, or 500-µm opening size. Data were collected from three independent experimental runs and are presented with a regression line and a 95% confidence interval band.

In general, the feeding device consists of three main components: a waterproof solid plane (e.g., the undersurface of a Petri dish), a nylon mesh sheet with an opening size of 50, 100, or 500 µm (#62-0866-38, #2-9566-05 or #2-9566-03; As One, Osaka, Japan) and a paraffin wax film (Parafilm M; Bemis, Neenah, WI, USA). First, a piece of the mesh sheet is placed on the undersurface of a polystyrene Petri dish flipped upside down (**Figure 1A**). Second, an appropriate volume (see below) of test solution or suspension is pipetted into the mesh sheet. Third, the entire mesh sheet is covered with a piece of Parafilm stretched to almost four times its original area to prevent evaporation of the liquid and to allow test arthropods to suck the underlying liquid by piercing the Parafilm membrane (**Supplementary Video S1**). Finally, the liquid-filled area of the mesh sheet is surrounded with a wetted Kimwipe (Nippon Paper Crecia, Tokyo, Japan) or an adhesive (Tangle B; Fuji Pharm, Tokyo, Japan) to form a feeding arena into which mites or aphids, respectively, can be placed. **Figures 1B, C** represent the assembled feeding devices for mites and aphids, respectively. A food dye (Brilliant Blue FCF; Fujifilm Wako Pure Chemical, Osaka, Japan) was used to visualize the distribution of liquid applied in the mesh sheet and to trace it in the digestive tracts of test arthropods. The structure of the feeding device and its use for oral administration are patent pending (Japanese Patent Application No. 2018-197157).

### Liquid Volume Required for the Feeding Device

To determine the volume of liquid required to fill the entire mesh sheet (4 cm^2^) with opening sizes of 50, 100, or 500 µm in the feeding device, we tested 10 to 160 µl of the 1% (w/v) blue dye solution. The test was conducted in three independent experimental runs for each volume and mesh opening size. The feeding devices were then scanned with an image scanner (GTX980; Seiko Epson, Suwa, Japan), and the proportion of the area filled with the blue dye solution was determined using an image processing program (ImageJ 1.52f).

### Time Required for *T. urticae* Feeding

Adult emergence of mites was synchronized as described previously (Suzuki et al., 2017a). To determine the time required for mite feeding in the mesh method, about 150 newly emerged adult *T. urticae* females were placed onto the feeding device using the nylon mesh sheet (2 cm^2^) with a 100-µm opening to which 40 µl of 1% (w/v) blue dye solution was added. The *T. urticae* females were allowed to feed on the solution for 1 to 4 h under standard laboratory conditions. Mite feeding was determined by the change of body color. The test was conducted in three independent experimental runs.

### dsRNA Synthesis

Total RNA was extracted from about 800 adult TSSM females frozen in liquid nitrogen with NucleoSpin RNA Plus (Macherey-Nagel, Düren, Germany) according to the manufacturer’s protocol. The quality and quantity of RNA were measured using a spectrophotometer (NanoPhotometer N60; Implen, Munich, Germany). cDNA was synthesized from 3 µg of total RNA using reverse transcriptase (SuperScript II Reverse Transcriptase; Thermo Fisher Scientific, Waltham, MA, USA) and an oligo (dT)_12–18_ primer (Thermo Fisher Scientific) according to the manufacturer’s protocol. cDNA was then stored at −30°C until use. Genomic DNA (gDNA) was extracted using a NucleoSpin Tissue extraction kit (Macherey-Nagel) and stored at −30°C. Using cDNA or gDNA as a template, specific primers targeting a 600-bp fragment of the *TuVATPase* gene (*tetur09g04140*) or a 382-fragment of the intergenic region (negative control [NC], genomic coordinates: scaffold 12, position 1690614–1690995) were used, respectively, for PCR amplification using DNA polymerase (Phusion High-Fidelity DNA Polymerase; New England Biolabs, Ipswich, MA, USA). Primers designed to amplify the DNA fragments of *TuVATPase* and NC are shown in **Table 1**. DNA fragments were then purified with NucleoSpin Gel and PCR Clean-Up Kit (Macherey-Nagel). The integrity of the purified DNA fragments was further confirmed with 2% (w/v) agarose gel (Agarose 21; Nippon Gene, Tokyo, Japan) electrophoresis, quantified with the spectrophotometer, and stored at −30°C until use. A template of 0.1 µg of each DNA fragment was used for RNA synthesis with an *in vitro* Transcription T7 Kit (Takara Bio, Kusatsu, Japan) in 1.5-ml centrifuge tubes according to the manufacturer’s protocol. After DNase I (Takara Bio) treatment for 30 min, RNA was denatured at 95°C for 5 min followed by slow cool-down to room temperature to facilitate dsRNA formation (Suzuki et al., 2017b). The dsRNA fragments (dsRNA-*TuVATPase* and dsRNA-NC) were purified by phenol-chloroform extract and precipitated with ethanol, quantified, and confirmed with the spectrophotometer and 2% (w/v) agarose gel, respectively.

**Table 1:** Primers used in this study for dsRNA production and real-time qRT-PCR analysis.

PCR amplification primers		
Primer name	Oligonucleotide sequence (5′ to 3′)^a^	Size (bp)
Tetur-VATP-F	GTTGCGGTGAGAGAGGTAATG	600
Tetur-VATP-R	GAAGAGGTACGAAATCTGGG	
Tetur-sc12-F	GCCCTCTCCTGGTTGTAAACTT	382^b^
Tetur-sc12-R	CGACCCCATCAGGCTATTGA	
**qPCR analysis primers**		
Primer name	Oligonucleotide sequence (5′ to 3′)	Primer efficiency
RP49 (tetur18g03590) F	CTTCAAGCGGCATCAGAGC	100.9%
RP49 (tetur18g03590) R	CGCATCTGACCCTTGAACTTC	
VATPase qPCR F	GGGTACCATCACATTCCTCG	103.3%
VATPase qPCR R	AATCGGTCTGGTTTGACGAAC	

^a^Primers for amplifying the DNA fragments for dsRNAs include the T7 promoter sequence (TAATACGACTCACTATAGGG) at the 5′ end.

^b^Negative control (NC) fragment (Suzuki et al., 2017b).

### Oral Delivery of dsRNA to *T. urticae*

Newly emerged adult *T. urticae* females were placed onto the feeding device (~30 mites/device) using 1 cm^2^ of nylon mesh sheet (opening size: 100 µm) to which 1 µg µl^−1^ of dsRNA-*TuVATPase* or dsRNA-NC and 1% (w/v) of blue dye solution had been added. The *T. urticae* females were allowed to feed for 24 h under standard laboratory conditions. Fed mites were transferred onto 1-cm-diameter kidney bean leaf discs (1 mite/disc), and the survivorship, fecundity, and dark-body phenotype previously reported in oral delivery of dsRNA-*TuVATPase* (Suzuki et al., 2017b) were observed with a stereomicroscope (SZ40; Olympus, Tokyo, Japan) for 6 days in the laboratory. The RNAi assay was conducted in three or four independent experimental runs.

### Real-Time Quantitative Reverse Transcription-PCR Analysis

Approximately 50 adult *T. urticae* females were collected at 2, 3, and 4 days after feeding on dsRNA-*TuVATPase* or dsRNA-NC, kept frozen in liquid nitrogen, and stored at −80°C until use. Total RNA was extracted using NucleoSpin RNA Plus (Macherey-Nagel), and single-stranded cDNA was synthesized by reverse transcription of total RNA using the High Capacity cDNA Reverse Transcription Kit (Thermo Fisher Scientific). Real-time quantitative reverse transcription-PCR (qRT-PCR) reactions were performed in three technical replicates with Power SYBR Green Master Mix (Thermo Fisher Scientific) on an ABI StepOnePlus Real-Time PCR System (Thermo Fisher Scientific). A gene encoding a ribosomal protein, *RP49*, was used as a reference gene (Suzuki et al., 2017b). Primers and amplification efficiencies for the reference gene (E_R_) and target gene (E_T_) are shown in **Table 1**. The threshold cycle (Ct) value was calculated by averaging three technical replicates. The expression value of the target gene (T) was normalized to the reference gene (R), and normalized relative quantity (NRQ) was calculated as follows: NRQ = (1 + E_R_)^CtR^/(1 + E_T_)^CtT^. The real time qRT-PCR analysis was conducted in three independent experimental runs.

### Oral Delivery of Synthetic Pesticides to *T. urticae*

The efficiency of the mesh method for bioassays was evaluated with two synthetic pesticides: abamectin (Agrimec; 1.8 g L^−1^ EC [emulsifiable concentrate], Syngenta Japan, Tokyo, Japan) and bifenazate (Mito-Kohne; 20 g L^−1^ FL [flowable], Nissan Chemical, Tokyo, Japan). Ten microliters of water-diluted abamectin (0.018 to 18 mg L^−1^ [=ppm]) or bifenazate (0.2 to 200 ppm) was applied to the feeding device using a 1-cm^2^ nylon mesh sheet with an opening size of 100 µm. Newly emerged adult *T. urticae* females (*n* = 29–92) were placed onto the feeding device, and the mortality was observed at 24 h and at 48 h for the treatment with abamectin and bifenazate, respectively. The pesticide assay was conducted in three independent experimental runs.

### Oral Delivery of Nanoparticles to *T. urticae*

To examine whether the mesh method can be used for oral delivery of nanoparticles, newly emerged adult *T. urticae* females were placed onto the feeding device (100 mites/device) using 1 cm^2^ of nylon mesh sheet (opening size: 100 µm) to which polystyrene fluorescent microspheres (diameter, 500 nm; Polysciences, Warrington, PA, USA) suspended in 1% (w/v) blue tracer dye solution was added. According to Bensoussan et al. (2018), mites can uptake 500-nm-diameter particles with their stylets, and the size cutoff is around 750 nm. After allowing mites to feed for 24 h, fluorescent images were taken with a digital camera (EOS Kiss X7, Canon, Tokyo, Japan) installed on a fluorescence stereomicroscope (M205FA; Leica Microsystems, Wetzlar, Germany) fitted with a GFP filter (395–455 nm excitation, >480 nm emission) with an exposure time of 5 s (ISO: 200). Bright-field images were taken using the same system without filters with an exposure time of 1 s (ISO: 200). The fluorescent microsphere assay was conducted in three independent experimental runs.

### Oral Delivery Assays in Other Spider Mites and an Insect

The mesh method was tested for the oral delivery of the blue tracer dye to other spider mite species (*T. evansi* and *T. kanzawai*) and to *A. gossypii* as a representative of sap-sucking insects. The same experimental procedure described for the *T. urticae* was used for *T. evansi* and *T. kanzawai* except that a 24-h fasting pre-treatment of newly emerged adult females prior to the assay was introduced in order to enhance the solution uptake. The starved *T. evansi* and *T. kanzawai* females (*n* = 50) were then introduced onto the feeding device and allowed to feed on 1% (w/v) blue tracer dye solution for 24 h under standard laboratory conditions. After 24 h, the number of mites to which the tracer dye was delivered was counted under a stereomicroscope. For *A. gossypii*, nylon mesh with an opening size of 500 µm was used in the feeding device, applying parameters preliminarily determined. The Parafilm of the feeding device for aphids was stretched less than that for spider mites, resulting in thicker film, in order to prevent them from cutting the Parafilm with their legs. In addition, the feeding arena was isolated with adhesive to prevent aphid escape (**Figure 1A**). Aphid nymphs and adults of various ages (*n* = 30–50) were placed onto the feeding device and allowed to feed on 2.5% (w/v) blue tracer dye solution for 24 h under the standard laboratory condition. After 24 h, the number of aphids to which the tracer dye was delivered was counted under a stereomicroscope.

### Data Analysis

All data analyses were performed with R v3.3.2 or v4.0.0 (R Core Team, 2016; R Core Team, 2020). Results for the relative area of distribution of the dye applied in the feeding device and the relative number of dye-fed mites are presented with a regression line and a 95% confidence interval band (R package: *ggplot2*). Survival curves were plotted with the Kaplan–Meier method (R function: survfit, package: *survival*). Differences in the survival curves between the dsRNA treatments were analyzed using the log-rank test (R function: survdiff, package: *survival*). Data normality and equality of variance in fecundity, cumulative proportion of dark phenotype mites, and relative quantity of *TuVATPase* gene expression were analyzed with the Shapiro–Wilk test (R function: shapiro.test) and *F*-test (R function: var.test). Differences in daily mite fecundity between the dsRNA treatments were statistically analyzed using the Wilcoxon–Mann–Whitney test (R function: wilcox.exact, package: *exactRankTests*). Arcsine square-root transformation was applied to normalize the cumulative proportion of mites with dark phenotype. Differences in the normalized proportional data and the relative quantity of *TuVATPase* gene expression between the dsRNA treatments were analyzed with a *t*-test (R function: t.test). Results for the daily fecundity and the cumulative proportion of mites with dark phenotype are presented as overlaid bee-swarm (R function: beeswarm, package: *beeswarm*) and box-and-whisker plots (R function: boxplot). The dose–response curves in the pesticide assay were generated with the two-parameter log-logistic function (R function: drm, R package: *drc*) and are represented with a 95% confidence interval band.

## Results

### Liquid Volume Required for the Feeding Device in the Mesh Method

The feeding device in which 4 cm^2^ of nylon mesh with a pore size of 50, 100, or 500 µm required 30, 40, or 160 µl (7.5, 10, or 40 µL cm^−2^) of liquid, respectively, to saturate the entire area (**Figure 1D**).

### Time Required for *T. urticae* Feeding in the Mesh Method

**Figure 2:**
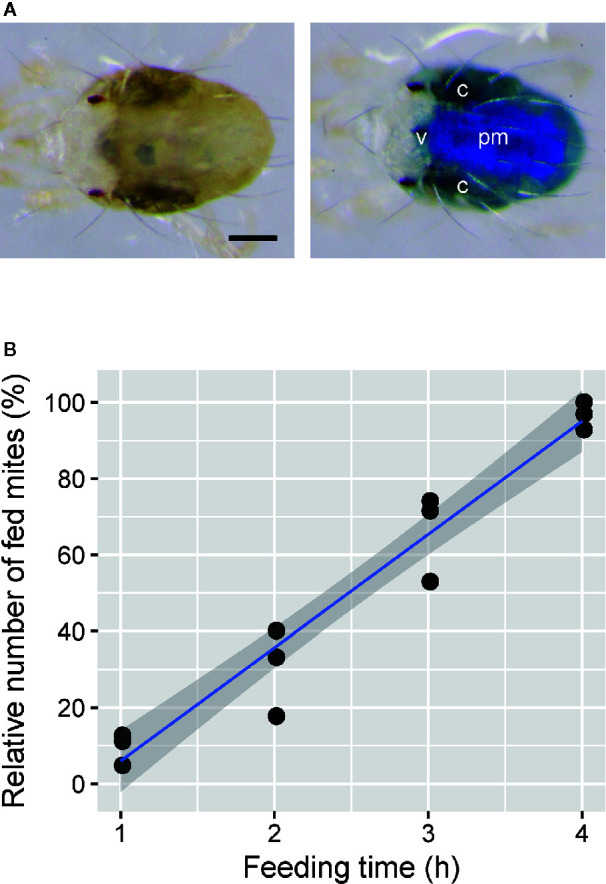
Efficiency of oral administration to *Tetranychus urticae* using the feeding device. **(A)** Adult females kept on the feeding device in which a 1% (w/v) blue tracer dye (Brilliant Blue FCF) was excluded (left) or included (right) at 25°C for 24 h after molting. C, caeca; v, ventriculus; pm, posterior midgut. Scale bar: 100 µm. **(B)** The relationship between the feeding time and relative number of fed mites. Newly molted adult females were placed onto the feeding device using a nylon mesh sheet (2 × 2 cm) with a 100-µm opening, and ingestion was determined by the change of body color. Data were collected from three independent experimental runs and are represented with a regression line and a 95% confidence interval band. *n* = 127–193.

Adult *T. urticae* females fed on blue tracer dye solution in the feeding device for 24 h showed blue color in the midgut (**Figure 2A**), which consists of the ventriculus, caeca, and posterior midgut (Bensoussan et al., 2018). In the fed mites, the blue color was most concentrated in the posterior midgut, consistent with the filtering of small molecules (<1 to 4 kDa) from the ventriculus to the posterior midgut (Bensoussan et al., 2018). However, blue dye was also visible in the caeca. The proportion of dye-fed mites increased over the feeding time (**Figure 2B**; **Supplementary Video S2**). The tracer dye was observably delivered to approximately 95% of mites after feeding for 4 h.

### Oral Delivery of dsRNA for RNAi of *TuVATPase*

**Figure 3:**
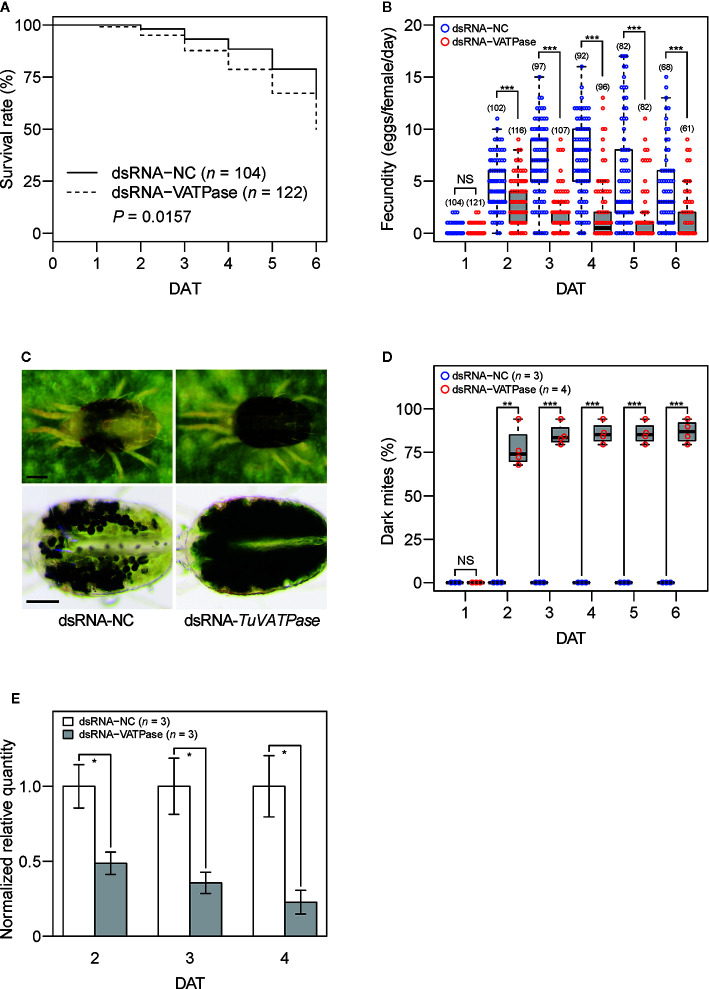
The effect of 1 µg µl^−1^ dsRNA-*TuVATPase* or dsRNA-NC (negative control) delivered *via* the mesh method on the survivorship, fecundity, and endogenous *TuVATPase* gene expression in adult *Tetranychus urticae* females at 25°C. **(A)** Survivorship of adult females for 6 days after treatment (DAT) with dsRNA-*TuVATPase* and dsRNA-NC. Survival curves were plotted by using the Kaplan–Meier method and compared by using the log-rank test. **(B)** Daily fecundity of adult females that survived after treatment with dsRNA-*TuVATPase* and dsRNA-NC. Data were represented by bee-swarm and box-and-whisker plots and compared by using Wilcoxon–Mann–Whitney tests (NS, *P* > 0.05; ***, *P* < 0.001). Values in parentheses indicate the number of surviving mites. **(C)** Mite body phenotype associated with dsRNA treatment. Lower photos are of mites soaked in a 50% (v/v) glycerol and 0.1% (v/v) Triton X-100 solution. Scale bar: 100 µm. **(D)** Cumulative frequency of dark-body phenotype observed after treatment with dsRNA-*TuVATPase* and dsRNA-NC. Data were represented by bee-swarm and box-and-whisker plots and compared by using a *t*-test after normalization with arcsine square-root transformation (NS, *P* > 0.05; **, *P* < 0.01; ***, *P* < 0.001). Data were collected from four and three independent experimental runs for treatments with dsRNA-*TuVATPase* (*n* = 4) and dsRNA-NC (*n* = 3), respectively. In each experimental run, 25 to 42 mites were used. **(E)** *TuVATPase* gene expression relative to the expression of *RP49* reference gene at 2, 3, and 4 days after treatment with dsRNA-*TuVATPase* and dsRNA-NC. Data were represented as mean ± SE and compared by using a *t*-test (*, *P* < 0.05). **(A, B, E)** Data were collected from three independent experimental runs. **(B, D)** In the box-and-whisker plots, the central line (second quartile, Q2) indicates the median, the distance between the box bottom (first quartile, Q1) and top (third quartile, Q3) indicates the interquartile range (IQR), and the whisker bottom and top indicate the minimum and maximum values, respectively. Outliers that are outside the range between the lower [Q1 − 1.5 × IQR] and upper limits [Q3 + 1.5 × IQR] are plotted outside of the IQR.

A significantly lower survivorship was observed in mites fed on dsRNA-*TuVATPase* than mites that fed on the control dsRNA (**Figure 3A**). The fecundity was significantly lower in mites fed on dsRNA-*TuVATPase* than in the control group at 2 to 6 days after treatment (**Figure 3B**). The dark-body phenotype that is associated with *VATPase* gene silencing in *T. urticae* (**Figure 3C**; Suzuki et al., 2017b; Bensoussan et al., 2020) was observed in around 80 and 90% of mites fed on dsRNA-*TuVATPase* at 2 days and at 3–6 days after treatment, respectively, whereas no mites showed the dark coloration in the control group even at 6 days after treatment (**Figure 3D**). The expression level of endogenous *TuVATPase* transcripts was significantly lower in the treatment group than in the control group at 2–4 days after treatment (**Figure 3E**).

### Oral Toxicity of Abamectin and Bifenazate

**Figure 4:**
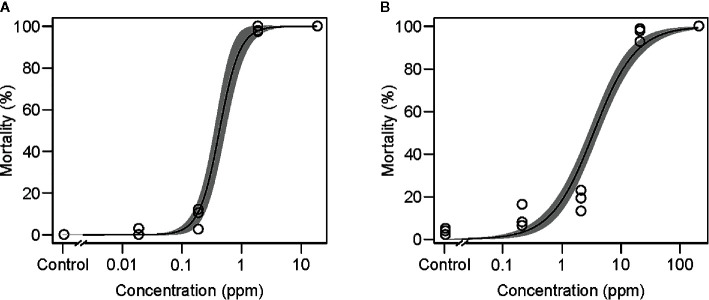
Effects of the acaricides **(A)** abamectin and **(B)** bifenazate delivered *via* the mesh method in adult *Tetranychus urticae* females at 25°C for 24 and 48 h, respectively. Data were collected from three independent experimental runs and presented with a regression curve and a 95% confidence interval band. In each experimental run, 25 to 92 mites were used.

More than 90% mortalities were observed in mites placed onto the feeding device and allowed to feed on >1.8-ppm abamectin (**Figure 4A**) and >20-ppm bifenazate (**Figure 4B**). The LC_50_ values were 0.43 ± 0.05 and 3.41 ± 0.35 ppm for mites ingesting abamectin and bifenazate, respectively. A rapid toxicity was observed in mites fed on 18-ppm abamectin and all mites tested were dead or dying within 2 h after placement on the feeding device (**Supplementary Video S3**).

### Oral Delivery of Particle Suspension

**Figure 5:**
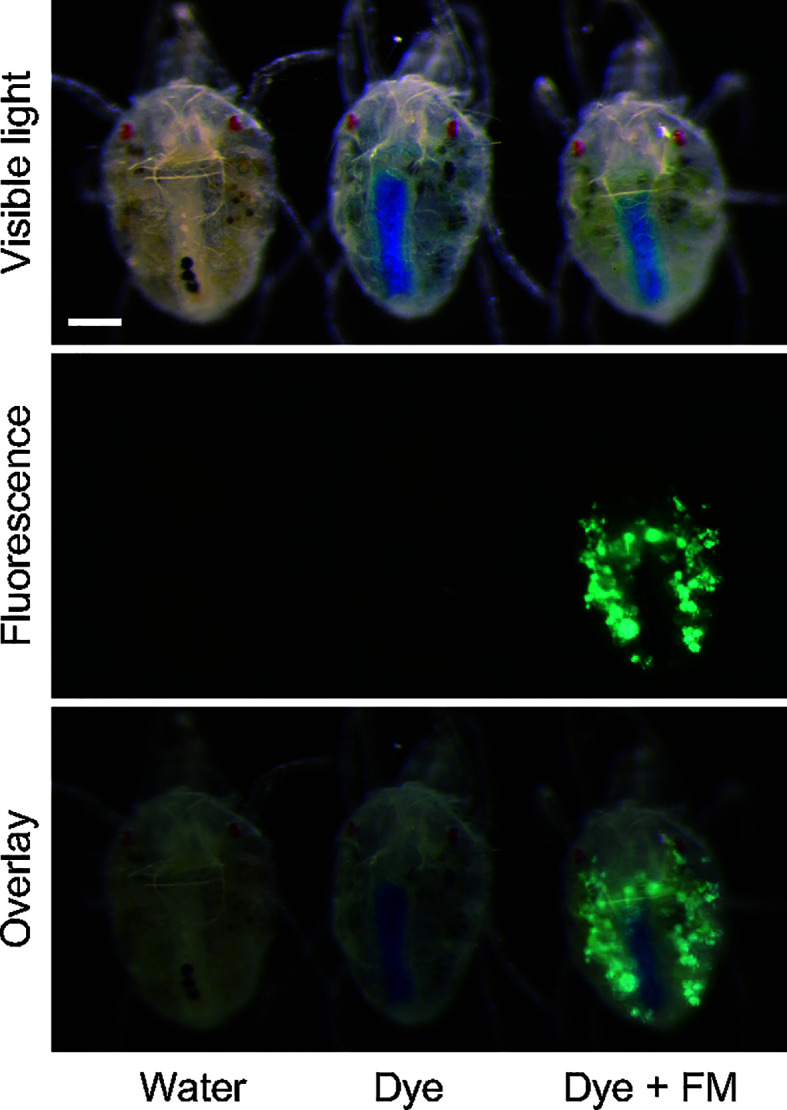
Oral delivery of fluorescent nanoparticles (diameter, 500 nm) to adult *Tetranychus urticae* females with the mesh method. Mites were allowed to ingest at 25°C for 24 h. In the control, water or 1% (w/v) blue tracer dye (Brilliant Blue FCF) solution was placed in the feeding device.

Fluorescent microspheres were delivered to approximately 90% of mites (*n* = 309) that were allowed to ingest 500-nm-diameter fluorescent microsphere suspensions for 24 h, and the fluorescent signal was observed in the ventriculus and caeca in the midgut (**Figure 5**).

### Liquid Delivery to Other Species

Unlike *T. urticae*, adult female *T. evansi* and *T. kanzawai* required prior starvation for 24 h to enhance their liquid uptake through the feeding device in the mesh method. After this pre-treatment, uptake of 1% (w/v) blue tracer dye solution was observed in more than 90% of *T. evansi* (*n* =105) and *T. kanzawai* females (*n* = 96) after feeding for 3 h (**Supplementary Figure S1A**). In the cotton aphid *A. gossypii*, uptake of 2.5% (w/v) blue tracer dye solution was observed in ~80% of nymphs and adults (*n* = 117) of various ages (**Supplementary Figures S1B, C**, **Supplementary Video S4**).

## Discussion

Resistance to conventional synthetic pesticides in arthropod herbivores imposes severe threats to the productivity of agricultural and horticultural crops. Therefore, it is important to develop new compounds or other strategies for arthropod pest control. However, the development of an effective compound requires numerous candidates to be screened with time-consuming bioassays. Thus, the development of a simple and effective method for the delivery of a wide range of compounds would support high-throughput screening of candidate molecules. Here, we reported a new method for the oral delivery of test compounds into spider mites and aphids. Our experiments demonstrate its applicability in environmental RNAi with exogenously supplied dsRNA and bioassays with synthetic pesticides and nanoparticles as a potential carrier of bio-active compounds in *T. urticae*.

The mesh method has a high efficiency of oral administration: >90% of mites ingested the tracer dye within 4 h (**Figure 2B**). Compared to the soaking method, where a 24-h ingestion time is provided (Suzuki et al., 2017a, b; Bensoussan et al., 2020), our method is time saving. In addition, the mesh method requires fewer resources, as 10 µl of liquid can be delivered to ~100 mites (*i.e.*, 0.1 µl/mite). This efficiency is quite high compared to previously reported methods for oral administration by soaking (1 µl/mite) (Suzuki et al., 2017a, b; Bensoussan et al., 2020), feeding on artificial diet filled in hemispherical Parafilm bubbles (3.3 µl/mite) (Suzuki et al., 2017a, b), and feeding on treated leaf discs (2–400 µl/mite) (Kwon et al., 2013; Suzuki et al., 2017b; Abouelmaaty et al., 2019). Furthermore, preparation of the feeding device is simple (**Figure 1A**). Hemispherical Parafilm bubbles used in the artificial diet method requires a custom-built vacuum device, as described by Jonckheere et al. (2016) and Suzuki et al. (2017a). All materials used in the mesh method are low-cost and general-purpose products that are likely available in most laboratories. Unlike the hemispherical Parafilm bubbles, the feeding device in the mesh method is flat like a leaf disc, making it easy to inoculate and maintain mites and allowing the inoculation of ~100 mites even in a limited area of 1 cm^2^, which enables the execution of the area-efficient bioassays. Moreover, the mesh method does not require post-treatment manipulation of mites, such as the rinsing and drying of soaked mites, which is the bottleneck of the soaking method (Suzuki et al., 2017b). Thus, mite handling is as easy as that on leaf discs, which enables time-efficient bioassays.

To evaluate the usefulness of the mesh method to deliver genetic materials, we tested environmental RNAi targeting the *TuVATPase* gene with exogenously supplied dsRNA in mites. The dark-body phenotype (**Figure 3C**) associated with RNAi targeting the *TuVATPase* gene was observed in approximately 90% of mites (**Figure 3D**), which was 2 to 3 times higher than that reported by Suzuki et al. (2017b) and comparable to that noted by Bensoussan et al. (2020), who tracked dsRNA ingestion using a tracer dye. In addition, we observed reductions in mite survivorship and fecundity by RNAi targeting the *TuVATPase* gene (**Figures 3A, B**). According to Suzuki et al. (2017b) and Bensoussan et al. (2020), a marked reduction in mite survivorship was observed at 6 to 10 days after treatment. In the present study, the reduction in mite survivorship was moderate when compared to these reports, partly because the observation period was limited to 6 days after treatment. Thus, an observation period of about 10 days should be used to accurately assess the lethal effect of environmental RNAi in mites. Although the dsRNA concentration used (1 µg µl^−1^) was higher than that used by Suzuki et al. (2017b) and Bensoussan et al. (2020) (20 to 320 ng µl^−1^), the reductions in mite survivorship and fecundity were comparable. Bensoussan et al. (2020) have reported no significant difference in the effects of RNAi targeting the *TuVATPase* and a subunit of coatomer protein complex (*TuCOPB2*) genes at dsRNA concentrations higher than 160 ng µl^−1^, which suggest that the lethal RNAi effect may have already been saturated at 1 µg µl^−1^ dsRNA. The *TuVATPase* transcript level reached >75% reduction in mites collected 4 days after treatment (**Figure 3E**). Although the reduction of the transcript abundance was 2 to 3 times higher than that reported in previous studies (Kwon et al., 2013; Suzuki et al., 2017b; Bensoussan et al., 2020), this might be due to the high concentration of orally administered dsRNA used in the present study. Optimizing the observation period (~10 days) and dsRNA concentration (>160 ng µl^−1^) in the mesh method should allow for high-throughput screening of candidate genes for RNAi-based *T. urticae* control.

The mesh method can also be used to evaluate the oral toxicity of synthetic pesticides to sucking arthropod herbivores. The LC_50_ values were 0.43 and 3.41 ppm in mites that ingested abamectin and bifenazate, respectively (**Figure 4**). These LC_50_ values are higher than those observed in leaf disc-sprayed bioassays (0.024 and 1.89 ppm, respectively) in which mites are exposed to both contact and oral toxicities (Khajehali et al., 2011). These results suggest that the mesh method is useful in evaluating the oral toxicity of pesticides and could be particularly applicable for testing the synergic effects between pesticides and RNAi targeting xenobiotic metabolic process genes.

Delivery of dsRNA *via* nanoparticles is a promising approach for enhancing the efficiency of environmental RNAi not only by increasing the stability of dsRNA but also by increasing the cellular uptake of dsRNA (Joga et al., 2016; Debnath and Das, 2018; Taning et al., 2020). The mesh method supported delivery of 500-nm-diameter particles to approximately 90% of mites (*n* = 309) within 24 h (**Figure 5**). Thus, the mesh method can be used to evaluate the significance of nanoparticles as dsRNA delivery vehicles.

The mesh method could be more widely used for delivering experimental solutions to other stylet-feeding arthropods. Although we were able to effectively deliver blue tracer dye to *T. evansi* and *T. kanzawai* by using the mesh method (**Supplementary Figure S1A**), unlike *T. urticae*, *T. evansi* and *T. kanzawai* required 24-h starvation before the experiment to enhance their feeding. It was also necessary to clear the gut of these mite species because their bodies are pigmented red, which obscures the blue color of the tracer dye. Therefore, a starvation period allowed *T. evansi* and *T. kanzawai* to excrete the dark digestive cells in the midgut as feces (Bensoussan et al., 2018), which helped the visualization of the tracer dye but might influence the outcome of follow-up experiments with dsRNA or acaricides. The impact of 24-h starvation on the follow-up experiments remains to be investigated. We also showed that the mesh method can be used for delivering the blue tracer dye solution to aphids (**Supplementary Figures S1B, C**). Although not tested in the present study, we hypothesize that the mesh method may also be used for delivering test compounds to blood-feeding insects such as bedbugs and mosquitos.

## Data Availability Statement

All datasets presented in this study are included in the article/**Supplementary Material**.

## Author Contributions

NG, VG, and TS planned the study. NG, MO, KS, SY, and FH performed the experiments and collected the data. NG and TS performed data analysis and visualization. NG, VG, and TS wrote the manuscript.

## Funding

This study was supported by a Japan Society for the Promotion of Science (JSPS) KAKENHI grant (18H02203) and Japan Science and Technology Agency OPERA grant (JPMJOP1833) to TS and was partly supported by the Institute of Global Innovation Research of Tokyo University of Agriculture and Technology to TS and VG. NG was supported by JSPS Invitational Fellowships for Research in Japan (L19542).

## Conflict of Interest

The authors declare that the research was conducted in the absence of any commercial or financial relationships that could be construed as a potential conflict of interest.

## Supplementary Material

Figure S1 Oral delivery of blue tracer dye to other sucking arthropod herbivores with the feeding device in the mesh method. **(A)** Adult females of the tomato red mite, *Tetranychus evansi*, and the Kanzawa spider mite, *Tetranychus kanzawai*, kept for 24 h on the feeding device filled with water (control) or 1% (w/v) blue tracer dye (Brilliant Blue FCF) solution. **(B)** Nymphs and adults of the cotton aphid, *Aphis gossypii*, kept for 24 h on the feeding device filled with water (control) or 2.5% (w/v) blue tracer dye solution. In adult aphids, almost half of individuals kept on the feeding device filled with 2.5% (w/v) blue tracer dye solution turned blue across the whole body, and others turned blue only in the digestive tract. **(C)** *Aphis gossypii* adult sucking the dye solution in the feeding device. The white arrowhead points at the stylets of the aphid. Scale bars: **(A)** 100 µm; **(B, C)** 500 µm.

Video S1 Preparation of the feeding device in the mesh method.

Video S2 Adult *Tetranychus urticae* females (*n* ≈ 50) sucking on 1% (w/v) blue tracer dye (Brilliant Blue FCF) solution in the mesh method feeding device for 1.5 h. The video is shown at 64× speed. Feeding arena: 5 × 5 mm.

Video S3 Adult *Tetranychus urticae* females (*n* = 50) sucking on 18-ppm abamectin in 1% (w/v) blue tracer dye (Brilliant Blue FCF) solution in the mesh method feeding device for 2.5 h. The video is shown at 128× speed. Feeding arena: 4 × 3 mm.

Video S4
*Aphis gossypii* nymphs sucking on 2.5% (w/v) blue tracer dye (Brilliant Blue FCF) solution in the mesh method feeding device for 16 min. The video is shown at 32× speed.
