# Supplementary figures and images for: A Leaf-Mimicking Method for Oral Delivery of Bioactive Substances Into Sucking Arthropod Herbivores
Source: Front Plant Sci. 2020 Aug 11;11:1218. doi: 10.3389/fpls.2020.01218 (PMC7431704; doi:10.3389/fpls.2020.01218)

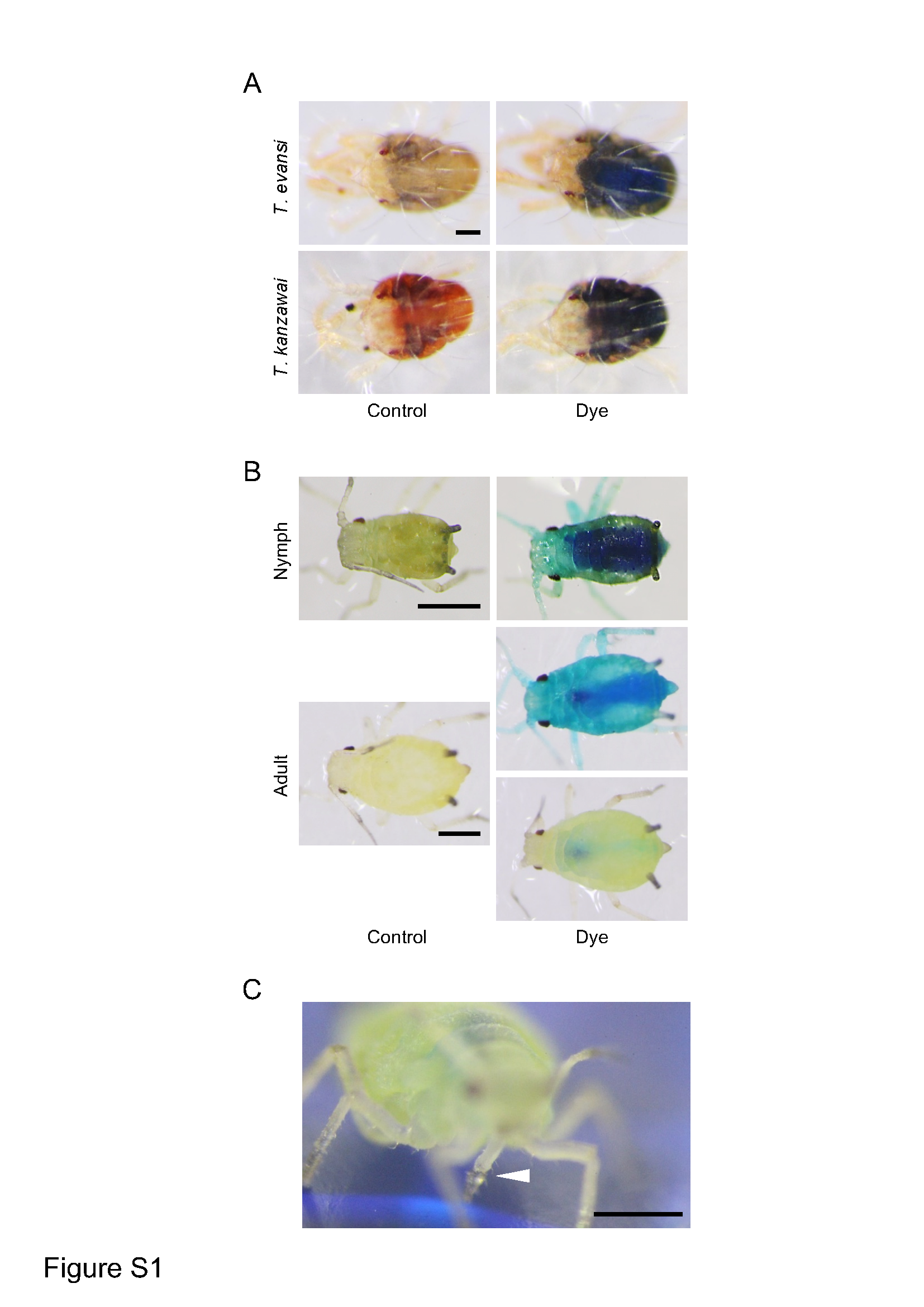

Supplement: Figure S1 — Oral delivery of blue tracer dye to other sucking arthropod herbivores with the feeding device in the mesh method. (A) Adult females of the tomato red mite, Tetranychus evansi, and the Kanzawa spider mite, Tetranychus kanzawai, kept for 24 h on the feeding device filled with water (control) or 1% (w/v) blue tracer dye (Brilliant Blue FCF) solution. (B) Nymphs and adults of the cotton aphid, Aphis gossypii, kept for 24 h on the feeding device filled with water (control) or 2.5% (w/v) blue tracer dye solution. In adult aphids, almost half of individuals kept on the feeding device filled with 2.5% (w/v) blue tracer dye solution turned blue across the whole body, and others turned blue only in the digestive tract. (C) Aphis gossypii adult sucking the dye solution in the feeding device. The white arrowhead points at the stylets of the aphid. Scale bars: (A) 100 µm; (B, C) 500 µm. [file Image_1.tif]
